# Expanding genotype–phenotype correlations in FOXG1 syndrome: results from a patient registry

**DOI:** 10.1186/s13023-023-02745-y

**Published:** 2023-06-12

**Authors:** Elise Brimble, Kathryn G. Reyes, Kopika Kuhathaas, Orrin Devinsky, Maura R. Z. Ruzhnikov, Xilma R. Ortiz-Gonzalez, Ingrid Scheffer, Nadia Bahi-Buisson, Heather Olson, the FOXG1 Research Foundation

**Affiliations:** 1 Invitae, San Francisco, CA USA; 2 Cedars Sinai, Los Angeles, CA USA; 3 MGH Institute of Health Professions, Boston, MA USA; 4 NYU Langone Health, New York, NY USA; 5 U.S. Food and Drug Administration, Silver Spring, MD USA; 6 Children’s Hospital of Philadelphia, Philadelphia, PA USA; 7 University of Melbourne, Melbourne, Australia; 8 Paris Descartes University/Neckers Hospital, Paris, France; 9 Boston Children’s Hospital, Boston, MA USA

**Keywords:** Patient registry, Genotype–phenotype association, Rare neurological diseases, Movement Disorder, Epilepsy

## Abstract

**Background:**

We refine the clinical spectrum of FOXG1 syndrome and expand genotype–phenotype correlations through evaluation of 122 individuals enrolled in an international patient registry.

**Methods:**

The FOXG1 syndrome online patient registry allows for remote collection of caregiver-reported outcomes. Inclusion required documentation of a (likely) pathogenic variant in *FOXG1*. Caregivers were administered a questionnaire to evaluate clinical severity of core features of FOXG1 syndrome. Genotype–phenotype correlations were determined using nonparametric analyses.

**Results:**

We studied 122 registry participants with FOXG1 syndrome, aged < 12 months to 24 years. Caregivers described delayed or absent developmental milestone attainment, seizures (61%), and movement disorders (58%). Participants harbouring a missense variant had a milder phenotype. Compared to individuals with gene deletions (0%) or nonsense variants (20%), missense variants were associated with more frequent attainment of sitting (73%). Further, individuals with missense variants (41%) achieved independent walking more frequently than those with gene deletions (0%) or frameshift variants (6%). Presence of epilepsy also varied by genotype and was significantly more common in those with gene deletions (81%) compared to missense variants (47%). Individuals with gene deletions were more likely to have higher seizure burden than other genotypes with 53% reporting daily seizures, even at best control. We also observed that truncations preserving the forkhead DNA binding domain were associated with better developmental outcomes.

**Conclusion:**

We refine the phenotypic spectrum of neurodevelopmental features associated with FOXG1 syndrome. We strengthen genotype-driven outcomes, where missense variants are associated with a milder clinical course.

**Supplementary Information:**

The online version contains supplementary material available at 10.1186/s13023-023-02745-y.

## Background

*FOXG1* is one of the earliest expressed genes in the developing brain, playing a critical role in specification and growth of the telencephalon through transcriptional repression of target genes [1]. *FOXG1* coordinates progenitor cell survival [2] and corticogenesis [3, 4], inhibits astrogenesis [5] and establishes the axonal tract of the corpus callosum [6]. Induced pluripotent neural precursor cells and neurons derived from individuals with FOXG1 syndrome reveal disorders of excitatory: inhibitory balance [7].

*P*athogenic variants in *FOXG1* cause a neurodevelopmental disorder characterized by severe developmental disability, microcephaly, epilepsy, and a complex hyperkinetic-dyskinetic movement disorder (FOXG1 syndrome, OMIM 613454) [8–13]. The incidence is estimated to be 2.8–3.5 per 100,000 live births [14]. Impairments across developmental domains are universally described, with functional hand use and expressive language greatly affected [11, 12]. Movement disorders are reported in > 90% of patients, characterized by dystonia, choreoathetosis, and orolingual dyskinesias [15]. The epilepsy phenotype in FOXG1 syndrome is heterogeneous, with variable ages of onset, seizure types, and response to medication [16]. Epilepsy occurs in up to 87% of affected individuals, with onset typically before 2 years of age [12, 13, 16]. Brain imaging in FOXG1 syndrome often demonstrates corpus callosum hypoplasia, frontal lobe predominant pachygyria, and delayed myelination [11, 13]. Through evaluation of 122 participants of the FOXG1 registry using a caregiver survey, we refine the phenotypic spectrum of FOXG1 syndrome and highlight novel genotype–phenotype correlations.

## Results

### Population and genotype characteristics

**Fig. 1:**
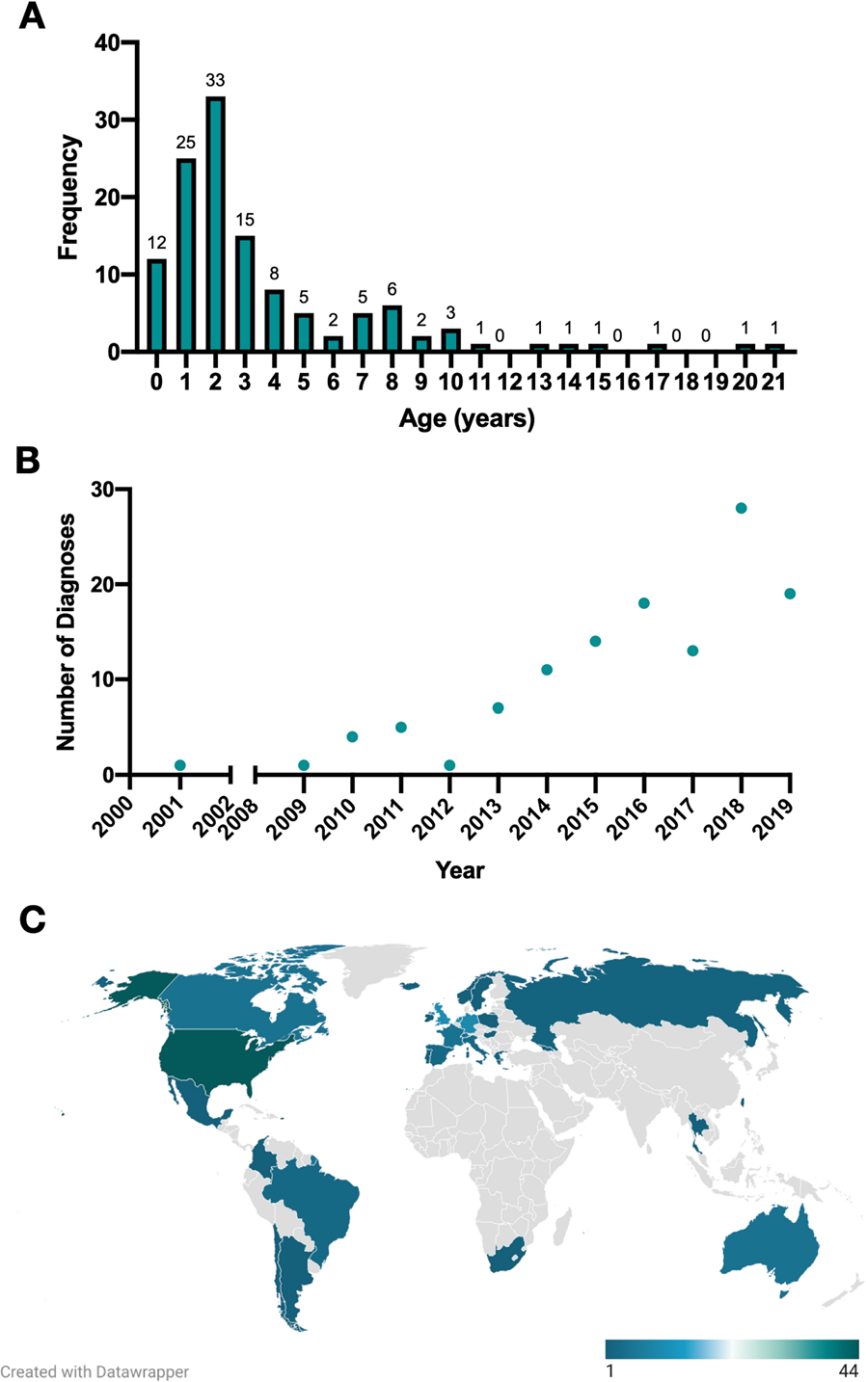
Inclusion criteria and demographic information for FOXG1 syndrome registry profiles. The age at diagnosis is presented in a histogram; approximately 75% of the FOXG1 syndrome cohort received a diagnosis before 5 years of age **(A)**. The number of diagnoses per year within the cohort appears to increase exponentially **(B)**. The FOXG1 syndrome patient registry was made available in English, French, German, Spanish, and Mandarin, facilitating international participation. Profiles were evaluated from individuals representing 29 countries **(C)**

Among 156 caregivers who enrolled in the FOXG1 registry, 122 provided a genetic test report that confirmed FOXG1 syndrome due to a likely pathogenic or pathogenic single nucleotide or copy number variant**.** Most participants were diagnosed before age 5 years (n = 93, 76%, Fig. 1A), with the number of new diagnoses generally increasing each year (Fig. 1B). Most diagnoses were achieved using clinical next generation sequencing, by gene panel or exome sequencing (Table 1). This international cohort comprised individuals from 29 countries (Fig. 1C). Demographic information grouped by genotypes with at least five individuals is presented in Table 1 (see Methods for additional detail)**.**

**Table 1:** Phenotypic and genotypic features of FOXG1 syndrome in 122 individuals

	Gene deletion	Missense	Nonsense	Frameshift	Total
n	19	39	18	38	122
Mean age in years (range)	7.05 (2 – 20)	6.05 (1 – 19)	5.50 (1 – 15)	7.47 (< 1 – 24)	6.67 (< 1 – 24)
Mean age at diagnosis in years (range)	2.53 (< 1 – 10)	3.67 (< 1 – 17)	2.44 (< 1 – 7)	4.71 (< 1 – 21)	3.61 (< 1 – 21)
*n (%)*					
Female	7 (36.8)	11 (28.2)	9 (50.0)	19 (50.0)	52 (42.6)
Variant Reported De Novo	17 (89.5)	33 (84.6)	16 (88.9)	29 (76.3)	99 (83.9)
Variant Confirmed De Novo	9 (47.4)	26 (66.7)	9 (50.0)	14 (36.8)	62 (52.5)
Parental Mosaicism	0 (0.0)	2 (5.1)	0 (0.0)	2 (5.3)	6 (5.1)
*Diagnostic test*					
Gene Panel					36 (35.3)
Exome Sequencing					35 (34.3)
aCGH					23 (22.5)
*FOXG1* sequencing					5 (4.9)
Karyotype					2 (2.0)
Family variant test					1 (1.0)
*n (%)*					
Epilepsy	13 (81.3)	16 (47.1)*	12 (70.6)	19 (57.6)	65 (61.3)
Movement Disorder	13 (81.3)	19 (55.9)	9 (52.9)	17 (51.5)	61 (57.5)
Epilepsy + Movement Disorder	11 (68.75)	9 (26.5)	8 (47.1)	11 (33.3)	42 (39.6)
Cortical Visual Impairment	12 (63.2)	9 (23.1)*	10 (55.6)	18 (47.4)	50 (41.0)
Strabismus	6 (46.2)	12 (42.9)	13 (81.3)	22 (78.6)	56 (63.6)
Hearing Loss	1 (5.3)	2 (5.1)	1 (5.6)	1 (2.6)	5 (4.1)
Mobility Device	16 (84.2)	23 (59.0)	12 (66.7)	28 (75.7)	84 (69.4)
Feeding Tube	11 (57.9)	7 (18.0)*	7 (38.9)	12 (32.4)	41 (33.9)
Autism	2 (12.5)	10 (32.3)	1 (6.3)	6 (19.4)	19 (19.2)

Demographic and clinical variables are presented for all participants in the FOXG1 registry (Total), as well as for genotypes with ≥ 5 individuals.

*and bolded values, significant difference compared to deletion genotype (*p* < 0.05), Fisher’s exact test with Bonferroni correction

**Fig. 2:**
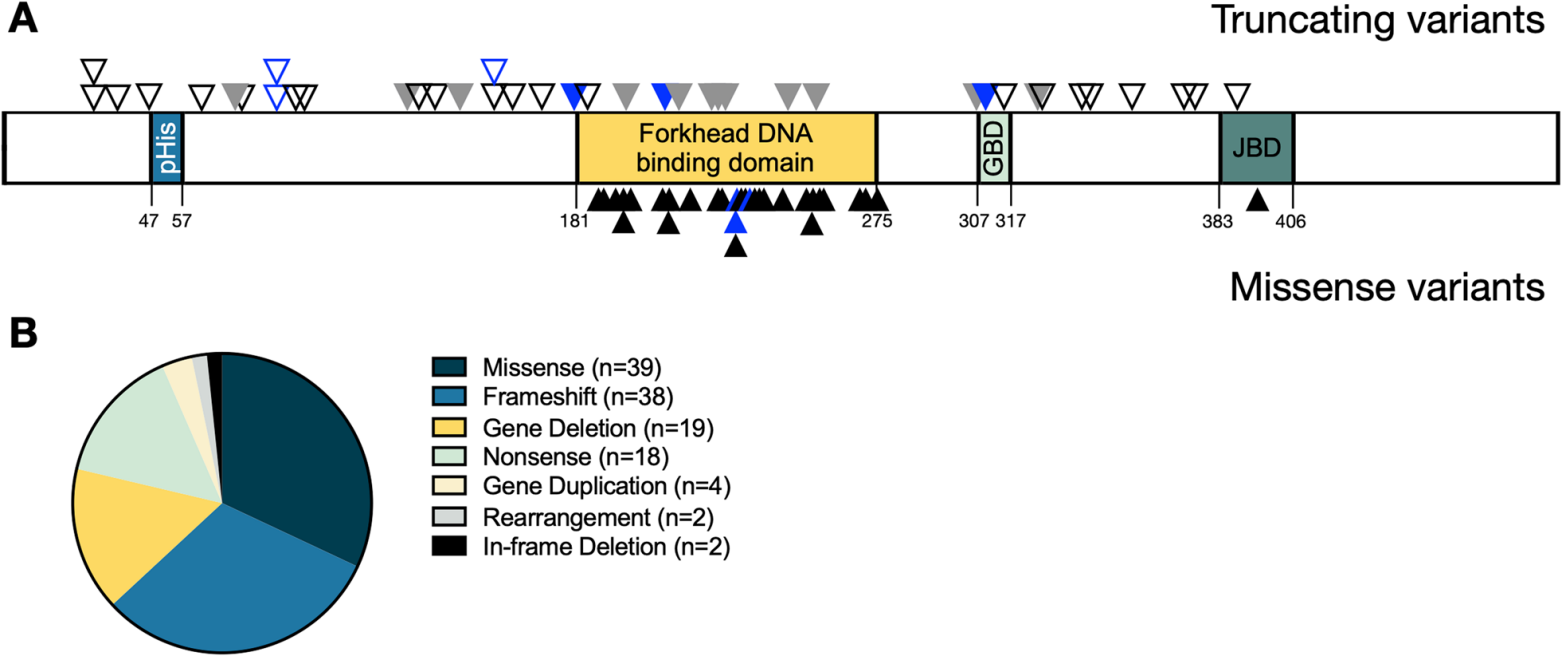
Spectrum of pathogenic variants reported in the FOXG1 syndrome patient registry. The distribution of reported pathogenic or likely pathogenic *FOXG1* missense, frameshift, and nonsense variants are depicted **(A)**. The majority of missense variants cluster in the conserved forkhead DNA binding domain, whereas frameshift and nonsense variants are dispersed throughout the coding sequence. Specific protein domains or characteristics have been outlined, including: a poly-histidine tract (pHis, amino acids 47–57), the forkhead DNA binding domain (amino acids 181–275), the Groucho-binding domain (GBD, amino acids 307–317), and a JARID-1B-binding domain (JBD, amino acids 383–406). Frameshift = ▽, recurrent frameshift = ▽, nonsense = ▼, recurrent nonsense = ▼, missense = ▲, recurrent missense = ▲. The frequency of unique genotypes across the 122 participants are shown in a pie chart **(B)**

We report 76 unique single nucleotide variants, 45 of which are novel and not previously described (Fig. 2, Additional file 1: Table S1). Frameshift and nonsense variants are distributed throughout the *FOXG1* coding sequence. Most causative missense variants in *FOXG1* localize to the conserved forkhead DNA binding domain (amino acids 181–275), including those in this cohort (38/39, 97%). One likely pathogenic missense variant (p.Ser393Trp) occurred outside of this domain. The p.Ser393 residue lies within a JARID-1B protein binding domain and could thereby impact FOXG1 function [17]. The FOXG1 registry identified 19 gene deletions and four gene duplications. Deletion size ranged from ~ 900 kb to ~ 6.8 Mb, encompassing an average of 9.6 genes (range 1–50). Duplication sizes ranged from ~ 2.2 Mb to ~ 14.7 Mb, encompassing an average of 21 genes (range 2–43). Whole gene duplications were included in aggregated analyses of the cohort, although the pathogenic mechanism remains unknown [18].

We found that missense (n = 39) and frameshift variants (n = 38) were most frequent. More than 80% of variants were reported by caregivers to be de novo; this was confirmed in approximately 50% with parental test reports. Parental mosaicism was documented in five families (six individuals total), which includes the only sibling pair observed to date in the FOXG1 registry. In all four individuals where parental testing results were available, maternal mosaicism was confirmed in blood through next generation sequencing.

### Developmental outcomes in FOXG1 syndrome

**Fig. 3:**
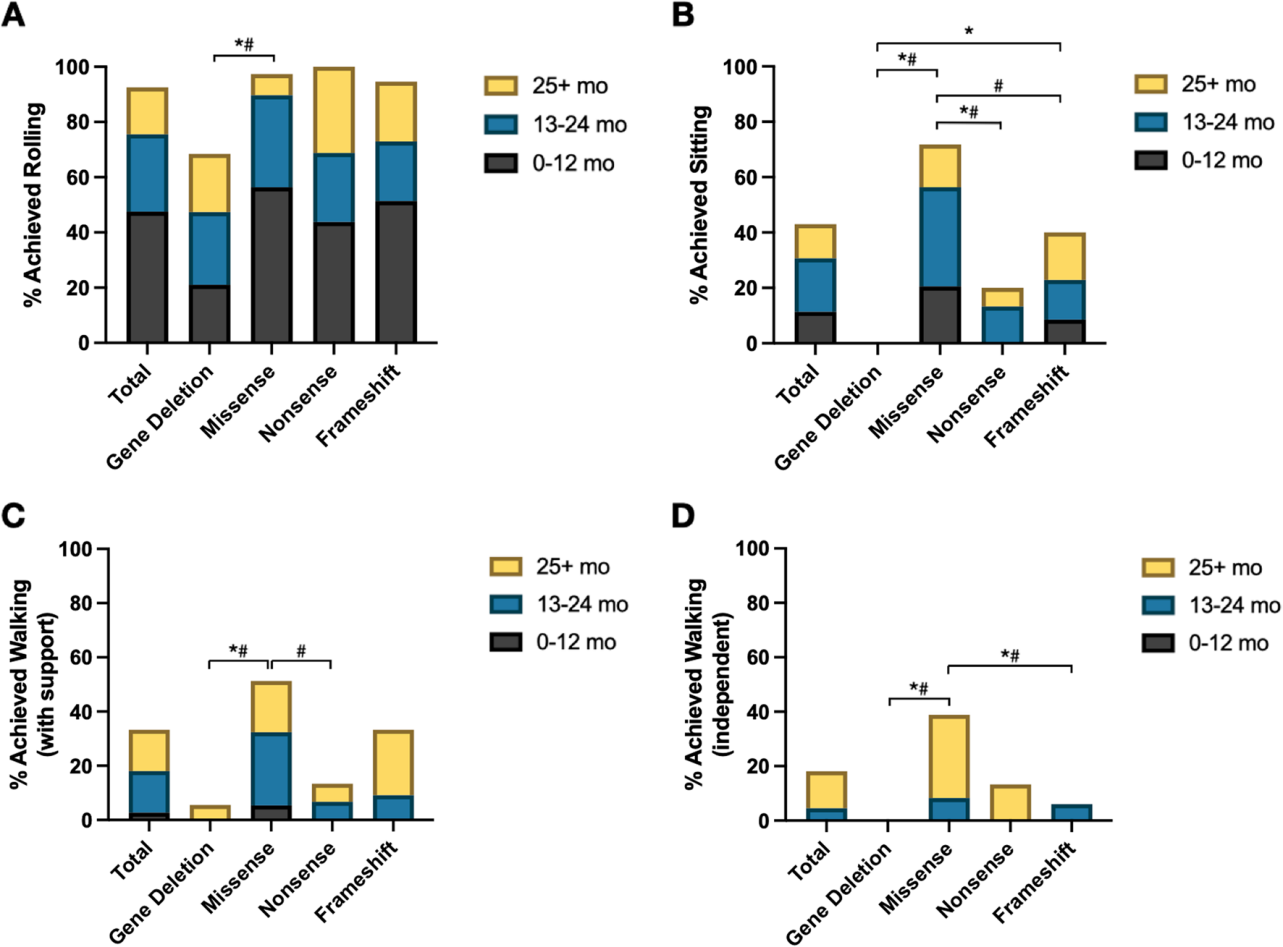
Gross motor milestones in FOXG1 syndrome. Caregivers in the FOXG1 syndrome patient registry reported whether a gross motor milestone was achieved as well as the reported age of attainment. Evaluated milestones include rolling (**A**), sitting independently (**B**), walking with support (> 12 months) (**C**), and walking independently (> 12 months) **(D)**. Milestone achievement was compared across genotypes using Fisher’s exact test with Bonferroni correction for multiple comparisons, *p < 0.05. The age of attainment was compared across genotypes using Kruskal–Wallis with Dunn’s test for multiple comparisons, #*p* < 0.05

**Fig. 4:**
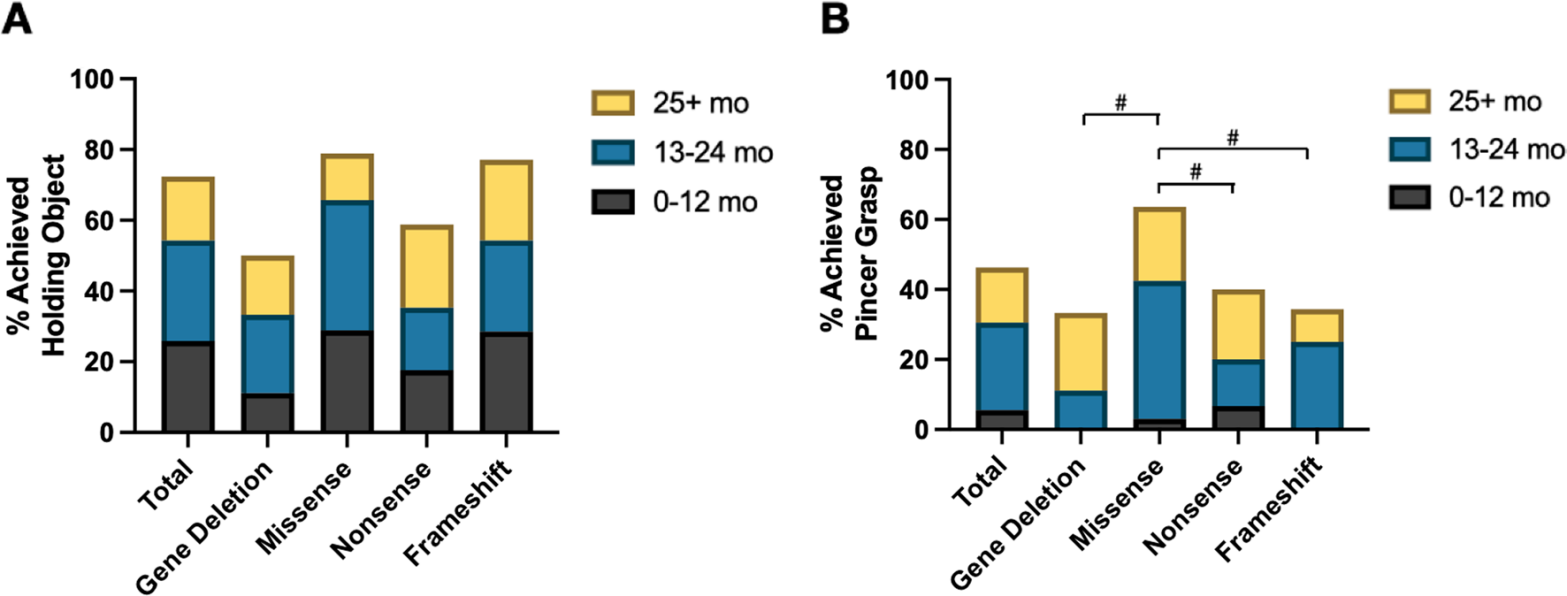
Fine motor milestones in FOXG1 syndrome. Caregivers in the FOXG1 syndrome patient registry reported whether a fine motor milestone was achieved as well as the age of attainment. Evaluated fine motor milestones include holding an object **(A)** and using a pincer grasp **(B)**. Milestone achievement was compared across genotypes using Fisher’s exact test with Bonferroni correction for multiple comparisons, *p < 0.05. The age of attainment was compared across genotypes using Kruskal–Wallis with Dunn’s test for multiple comparisons, # *p* < 0.05

Caregivers documented if a developmental milestone was achieved and assigned age of milestone attainment to one of three epochs: 0–12 months, 13–24 months, and 25 + months. There were no significant differences in age amongst the four genotype groups used in this analysis. In those individuals who had any developmental outcomes reported by the caregiver (n = 119), gross motor milestones were attained as follows: 92% rolled (either back-to-front or front-to-back, Fig. 3A), 43% sat independently **(**Fig. 3B**)**, 33% walked with support **(**Fig. 3C**)**, and 18% walked independently **(**Fig. 3D**)**. Across most gross motor milestones, a higher percentage of individuals with missense variants achieved milestones compared to those with frameshift or nonsense variants and gene deletions. Those with missense variants were more likely to sit independently (73%) than individuals with gene deletions (0%, *p* < 0.0001) or nonsense variants (20%, *p* = 0.0008). Similarly, for walking with support or independently, missense variants (53% and 41%) were associated with greater attainment when compared to gene deletions (6%, *p* = 0.0009 and 0%, *p* = 0.002), and frameshift variants for independent walking (6%, *p* = 0.0014). For fine motor milestones in the combined cohort, 73% could hold an object **(**Fig. 4A**)** and 46% achieved a pincer grasp **(**Fig. 4B**)**. The distribution of age of attainment across these epochs differed significantly between genotypes for rolling, sitting, walking (with/without support), and use of a pincer grasp **(**Fig. 3**, **Fig. 4**)**.

**Fig. 5:**
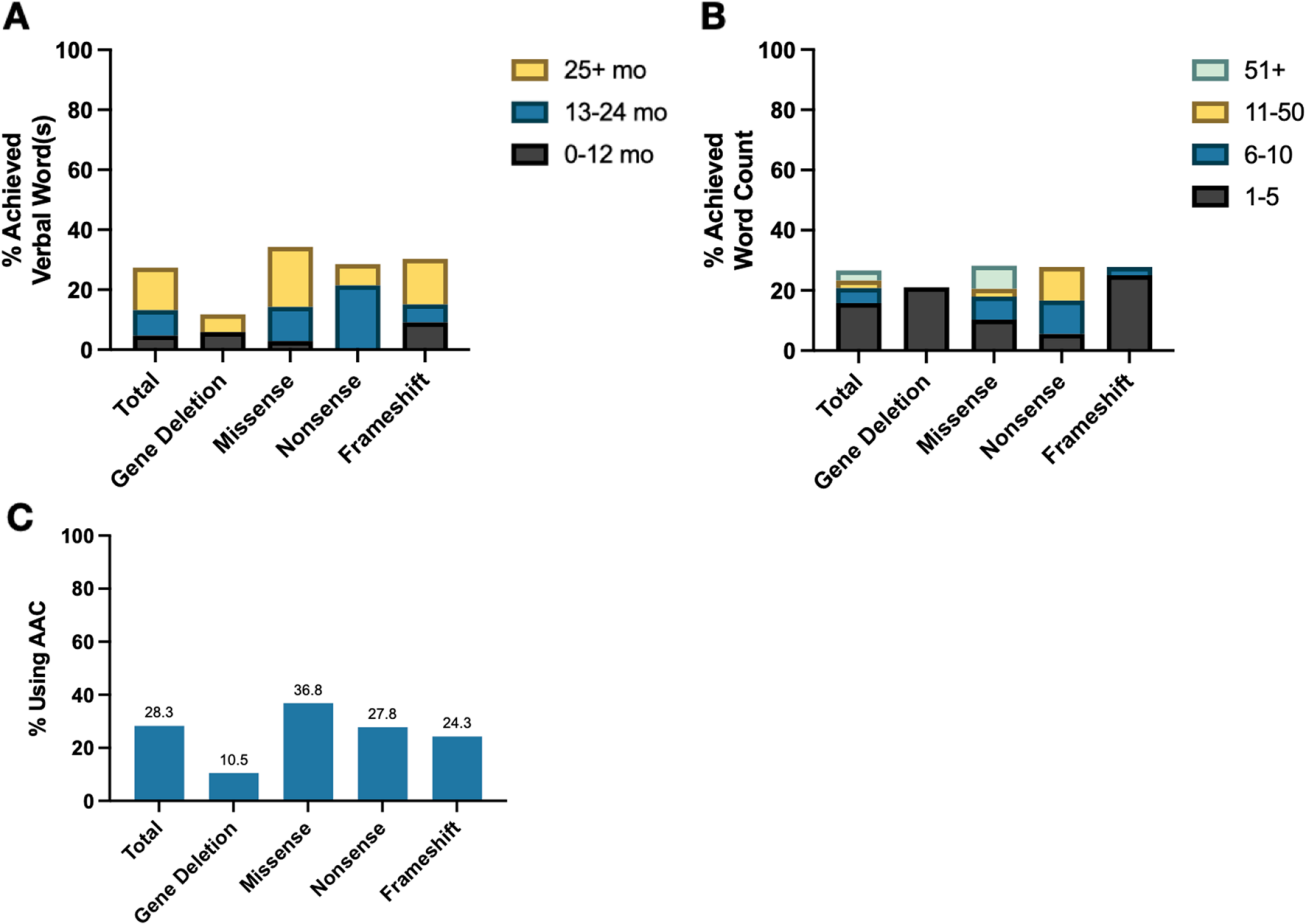
Language milestones in FOXG1 syndrome. Caregivers in the FOXG1 syndrome patient registry reported whether a language milestone was achieved as well as the reported age of attainment. To assess expressive language use in FOXG1 syndrome, caregivers reported the use of verbal words (> 12 months) (**A**), the number of verbal words used (**B**), and whether an AAC device was employed (**C**)

Expressive language was evaluated by determining 1) whether the participant spoke words **(**Fig. 5A**)**, 2) the total number of single words spoken **(**Fig. 5B**)**, and 3) use of an augmentative and alternative communication (AAC) device **(**Fig. 5C**)**. Overall, 27% of individuals spoke at least one word (n = 29); of these, most spoke their first word after age 25 months (n = 15, 52%) and used a maximum of 1–5 single words (n = 19, 66%). Speaking single words, the age of attainment, and the number of single words used did not differ across genotypes. Notably, three individuals with a de novo missense variant had ≥ 50 single words (p.Asn232Ser, p.Gly271Ser, and p.Ala188Glu). An AAC device was used by 28% of participants, including 18 who were non-verbal. No genotype significantly correlated with AAC use.

Caregivers were queried about developmental regression, which is not classically associated with FOXG1 syndrome. At least one episode of regression was reported for 68% of participants. Of those who provided additional detail, gross motor milestones were most commonly impacted: rolling (n = 6, 55%), sitting independently (n = 3, 27%), and walking independently (n = 1, 9%). Other affected developmental skills included grasping, head support, and social smile. Most caregivers could not identify a trigger for regression (n = 78, 94%). In five cases, a preceding event was recognized, such as a seizure (n = 4) or illness (n = 1).

Participants without epilepsy were more likely to sit independently (59% vs. 37%, *p* = 0.021), walk independently (30% vs. 14%, *p* = 0.044), and use a pincer grasp (61% vs. 42%, *p* = 0.041) compared to those with epilepsy. As the frequency of epilepsy in FOXG1 syndrome also varied between genotypes, we could not determine whether seizure history independently drives developmental outcomes.

### Behavioral problems in FOXG1 syndrome

Challenging behaviors were reported in 55% of the cohort (n = 66); compared to nonsense variants, those with missense variants were more likely to report behavioral problems (67% vs. 33%, *p* = 0.007). Autism spectrum disorder was diagnosed in 19% of individuals aged ≥ 3 years (n = 19); the frequency ranged from 6% in those with nonsense variants to 32% in those with missense variants but was not significantly different across genotypes. The higher rate of behavioral challenges and autism spectrum disorder in those with missense variants may reflect their higher levels of function.

### Epilepsy in FOXG1 syndrome

**Fig. 6:**
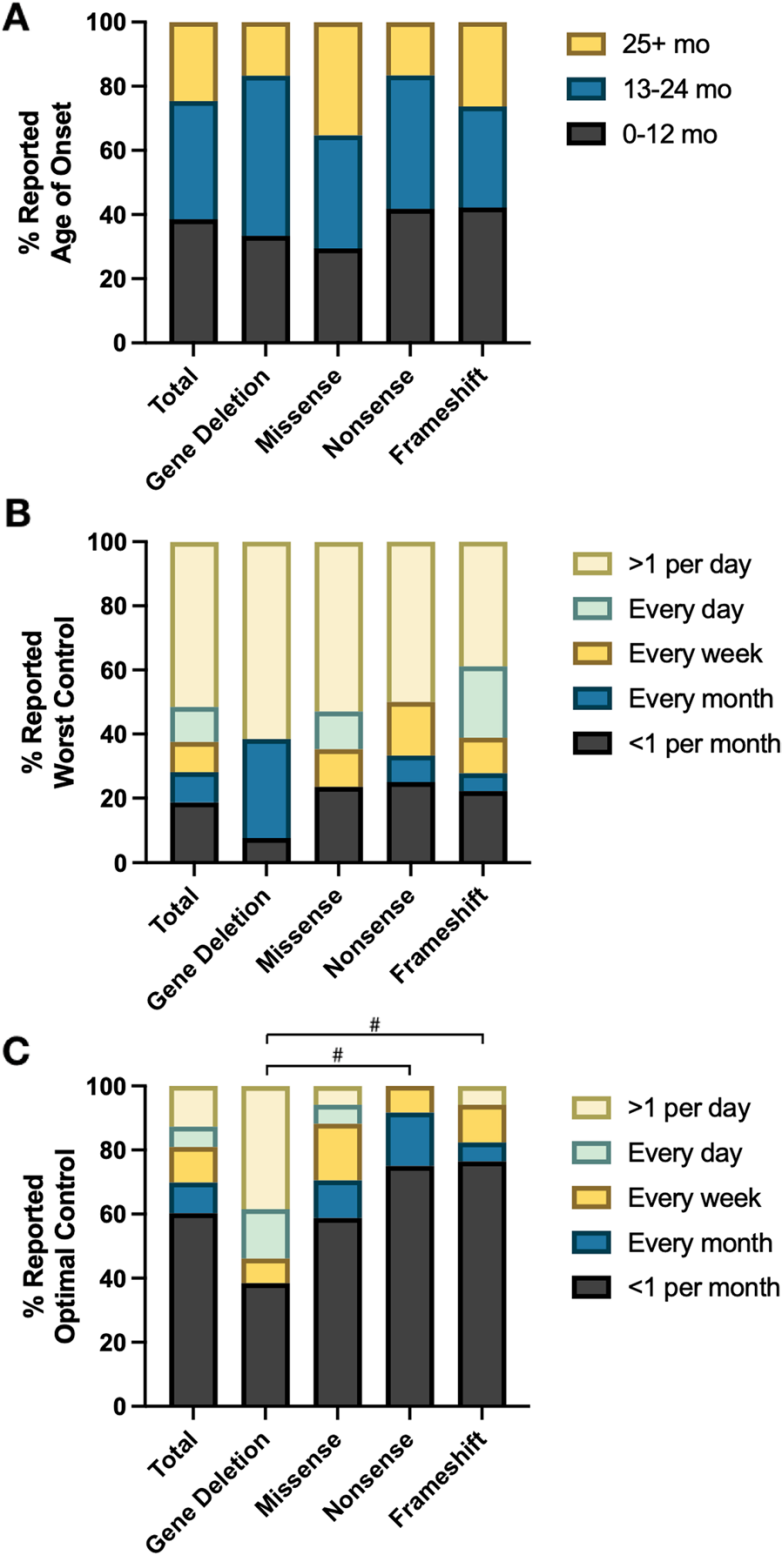
Epilepsy characteristics reported in FOXG1 syndrome. For those individuals with a reported history of epilepsy, caregivers were asked to describe the epilepsy phenotype in their child or ward as part of the FOXG1 syndrome patient registry. Proportions of age range for seizure onset are depicted (**A**) in addition to distributions of seizure frequency at worst (**B**) and optimal (**C**) seizure control. Comparisons between genotypes were evaluated using Kruskal–Wallis test with Dunn’s test to correct for multiple comparisons, #*p* < 0.05

Seizures occurred in 61% of participants (n = 65, Table 1). Individuals with missense variants were less likely to have seizures (47%) compared to those with a gene deletion (81%, *p* = 0.0007). In those individuals with a reported history of epilepsy, seizure onset most often occurred before age 25 months (n = 48, 75%; Fig. 6A). Multiple seizure types were reported, including tonic (n = 42, 66%), absence (n = 38, 59%), clonic (n = 31, 48%), myoclonic (n = 30, 47%), tonic–clonic (n = 29, 45%), infantile spasms (n = 13, 20%), focal (n = 8, 13%), and gelastic (n = 1, 2%). Twenty-six (42%) experienced a prolonged seizure lasting more than 15 min.

Caregivers of individuals with a reported history of epilepsy were asked to describe seizure frequency at the participant’s best and worst level of control. Overall, > 60% of caregivers reported at least daily clinical seizures when epilepsy was poorly controlled, which did not differ across genotypes (Fig. 6B). At best control, the frequency of daily seizures ranged from 0% in those with nonsense variants to 54% (n = 7) in those with gene deletions (Fig. 6C). At the time of best control for each individual, seizure frequency was greater in those with gene deletions compared to frameshift (*p* = 0.034) or nonsense variants (*p* = 0.049).

Seizure freedom (with or without medication) was reported in 31% (n = 20) of individuals at the time of enrollment, ranging from 8% in those with a gene deletion (n = 1) to 44% in those with a missense variant (n = 7).

Twenty-nine anti-seizure medications were used in this cohort, most often levetiracetam (n = 31, 58%), clobazam (n = 19, 36%), valproate (n = 18, 34%), clonazepam (n = 9, 17%) and lamotrigine (n = 9, 17%). The five medications most frequently endorsed by caregivers as effective were: levetiracetam (n = 14, 36%), valproate (n = 10, 26%), clobazam (n = 8, 21%), clonazepam (n = 3, 8%), and oxcarbazepine (n = 3, 8%). Seven individuals tried the ketogenic diet or modified Atkins diet to treat epilepsy, and three individuals had a vagus nerve stimulator implanted. Of those who reported using a therapeutic diet or device, only one endorsed the ketogenic diet as an effective treatment. No single medication was commonly reported by caregivers to exacerbate seizures.

### Movement disorders in FOXG1 syndrome

A movement disorder was present in 58%, although 21% of caregivers reported uncertainty. Participants with movement disorders were significantly older than those without (mean ages 8.4 vs. 5.9 years), suggesting that movement disorder may emerge with age or is less commonly recognized in younger children. Reported movement disorders included: spasticity (n = 17, 29%), chorea/athetosis (n = 17, 29%), hyperkinesis (n = 12, 21%), ataxia (n = 5, 9%), myoclonus (n = 5, 9%), tremor (n = 2, 3%), and dyskinesia (n = 2, 3%). Approximately 29% used a medication to treat the movement disorder, with 13 medications described. The most commonly used were baclofen (n = 5, 28%), tetrabenazine (n = 3, 17%), clonazepam (n = 3, 17%), trihexyphenidyl (n = 2, 11%), and clonidine (n = 2, 11%).

### Health status of individuals with FOXG1 syndrome

Cortical visual impairment (CVI) was reported in 41% (n = 50) and strabismus in 64% (n = 56). Of those with strabismus, 34% (n = 19) underwent surgical correction. Approximately 70% (n = 84) of participants required an assistive device for stability or ambulation, consistent with their limited attainment of gross motor skills. Due to risk of aspiration and/or feeding difficulties, a feeding tube was placed in 34% (n = 41) of this cohort. Those with missense variants were less likely to require a feeding tube (18% vs. 58%, *p* = 0.0053) or have CVI (23% vs. 63%, *p* = 0.0019) compared to participants with a gene deletion.

Caregivers reported current medications to highlight additional symptoms requiring intervention. Approximately 32% (n = 38) of participants used a laxative and/or proton pump inhibitor or antacid. A sleep aid was used by 22% (n = 26), including: melatonin, clonidine, and chloral hydrate. Sleep disruption was reported in 55% (n = 66) of participants. Additional medications used for other comorbidities in this cohort included gabapentin, glycopyrrolate, cannabidiol, and risperidone.

### Retaining the forkhead DNA binding domain confers a milder phenotype

As *FOXG1* is encoded by a single exon, frameshift and nonsense (truncating) variants should escape nonsense-mediated decay. We sought to determine whether retaining the forkhead DNA binding domain attenuates clinical severity. Individuals with N-terminal truncating variants (amino acids 1–275, n = 44) were compared to those with truncating variants that preserve the forkhead DNA binding domain (amino acids 276 + , n = 12). For all gross and fine motor skills evaluated, individuals with a C-terminal truncating variant were more likely to attain a given skill when compared to those with an N-terminal truncating variant; this only achieved statistical significance for pincer grasp (*p* = 0.023). No significant difference between presence of epilepsy or movement disorder was detected between the two groups. Across other variables, autism spectrum disorder was more common in those with C-terminal variants (44%) versus N-terminal variants (8%, *p* = 0.018).

## Discussion

FOXG1 syndrome is a rare genetic neurodevelopmental disorder that was originally described as “Rett syndrome, congenital variant” [8]. Through evaluation of a large cohort of 122 children and adults with likely pathogenic or pathogenic variants in *FOXG1*, we build upon previous work that establishes FOXG1 syndrome as a unique and recognizable disorder [12]. Further, we demonstrate that the clinical features and severity of FOXG1 syndrome may reflect the underlying genotype. We found that individuals with gene deletions show a more severe phenotype, with fewer achieving developmental milestones, more severe epilepsy, and need for more frequent medical intervention. This was most evident for epilepsy, with > 50% of participants with gene deletions reporting daily seizures, even at best control. As most individuals with a gene deletion had large copy number variants, the more severe presentation likely reflects involvement of additional genes and noncoding regulatory elements.

In contrast, *FOXG1* missense variants were associated with better developmental and health outcomes. They were more likely to attain gross motor milestones compared with other genotypes, such as independent walking, and had the lowest frequency of epilepsy. Other variables support a milder disease course, with reduced frequency of CVI, feeding tube, and use of a mobility device. The role of FOXG1 as a transcriptional repressor throughout early brain development is well characterized; however, DNA binding-independent functions have been established [19]. It is possible that missense variants within the forkhead DNA binding domain allow for residual FOXG1 function.

In addition to genotype–phenotype correlations, we also explored whether presence of epilepsy affected outcomes in FOXG1 syndrome. We observed that individuals without epilepsy were significantly more likely to achieve specific motor milestones as compared to those with epilepsy. However, we also observed that those genotypes associated with the highest frequencies of developmental milestone attainment had the lowest reported incidence of epilepsy. It is possible that a combinatorial effect of genotype and epileptic activity contribute to attainment of motor milestones in FOXG1 syndrome. Additional work is required to delineate the independent contributions of these two variables.

Two base pairs in the *FOXG1* coding sequence are particularly susceptible to variation. The c.460dupG frameshift variant (p.Glu154Glyfs*301) is commonly reported in FOXG1 syndrome. The c.256dupC (p.Gln86Profs*35) and c.256delC (p.Gln86Argfs*106) variants are also frequently identified. We did not find that these variants were associated with distinct phenotypes. Their presentation was consistent with other frameshift variants. In support, Seltzer et al*.* showed that the c.460dupG variant was associated with a similar epilepsy phenotype compared to other variants [16].

Two large cohorts of FOXG1 syndrome have been published: a prospective study of 45 affected individuals as well as a combined cohort of 83 novel and previously reported cases [12, 13]. The FOXG1 syndrome patient registry cohort closely mirrors that described by Mitter et al. with respect to developmental and epilepsy outcomes [12]. Both published cohorts use distinct clinical scores to evaluate global severity in FOXG1 syndrome. As observed in the FOXG1 syndrome patient registry, missense variants and C-terminal truncating variants are associated with milder disease in comparison to other genotypes [12, 13]. In aggregate, these studies confirm that those with N-terminal truncating variants and gene deletions are most severely affected. Of interest, the FOXG1 syndrome patient registry cohort demonstrates a lower frequency of epilepsy, with 61% of individuals having seizures compared to a published range of 68–87% [11–13, 15, 16]. Similarly, the frequency of movement disorder in this cohort (58%) is lower than previously described (90–100%) [11–13, 15]. The lower frequencies of epilepsy and movement disorder may reflect our broad recruitment strategy.

The primary limitation of caregiver responses in this registry is lack of direct clinical assessment and medical interpretation. We attempted to improve accuracy by tailoring the registry questionnaire to data elements familiar to caregivers, specifically excluding features such as electroencephalogram or brain imaging findings, which require additional expertise to interpret. Age of onset for core clinical features were collected in bins to minimize the impacts of recall bias. The registry was developed using international recommendations to promote data accuracy and utility [20, 21]. Rare disease registries leverage caregiver knowledge to expand clinical phenotypes and establish genotype–phenotype correlations [22, 23]. Across other disease groups, caregivers are reliable reporters, with high concordance observed between registry data and medical record documentation [24]. The electronic portal expands participant access for detailed analysis of large cohorts in rare diseases. This approach is complementary to ongoing clinical natural history data collection and is critical to guide clinical endpoint development.

## Conclusion

Our international patient registry of 122 individuals with FOXG1 syndrome further refines the clinical spectrum in children and adults. By leveraging caregiver reported data, we characterize genotype–phenotype correlations within FOXG1 syndrome. Our results confirm that *FOXG1* missense variants are associated with a milder disease course. Further work is required to establish additional modifying effects that contribute to variability in the FOXG1 syndrome phenotype.

## Methods

### FOXG1 syndrome patient registry

The FOXG1 syndrome patient registry is an online international platform available in English, French, German, Spanish, and Mandarin [25]. Parents or caregivers uploaded genetic testing reports for review by a genetic counselor (EB) to confirm a pathogenic or likely pathogenic variant in *FOXG1*. When a test report was unavailable, written documentation from a physician was accepted. Data presented were from a questionnaire administered to registry members between February 2019 and August 2019.

### Genetic variant review and interpretation

All single nucleotide variants were classified according to the American College of Medical Genetics and Genomics criteria [26]. Frameshift and nonsense variants are presumed to create a truncated protein through introduction of a premature stop codon; all frameshift and nonsense variants were presumed pathogenic, as loss-of-function variants cause FOXG1 syndrome [8]. Variants correspond to the only RefSeq transcript for *FOXG1*, NM_005249. Deletion and duplication breakpoints were mapped if coordinates and reference genome build were provided. Parental reports were available for 62 (53%) individuals. Parental mosaicism was confirmed in four individuals and presumed in two siblings carrying the same pathogenic variant.

### Questionnaire administration

Caregivers received a questionnaire about the core features of FOXG1 syndrome, including developmental capabilities, epilepsy, movement disorder, and complex medical management. Questionnaire development was informed by the authors’ clinical experience and literature review and was a collaborative process with parent advocates. When a history of movement disorder or epilepsy was reported, additional details were requested (e.g., seizure frequency, use of pharmacologic or surgical intervention).

### Statistical analysis

We stratified participants by genotype into seven groups: [1] whole gene deletion, [2] whole gene duplication, [3] rearrangement, [4] missense, [5] nonsense, [6] frameshift, and [7] in-frame deletion. Only groups with ≥ 5 individuals were included in statistical analyses to identify genotype–phenotype correlations. For developmental milestones (i) walk (with/without support) and (ii) use of verbal speech, analyses were limited to those > 12 months of age. Similarly, for autism spectrum disorder diagnosis, analyses were limited to those ≥ 3 years of age. Nonparametric tests evaluated differences between genotypes, including Fisher’s exact test with Bonferroni correction for multiple comparisons, Kruskal–Wallis test with Dunn’s correction for multiple comparisons, ANOVA with Tukey’s post-hoc test, and Mann–Whitney U test; corrected *p* < 0.05 was statistically significant. A one-tail t-test evaluated for significant differences in age between two compared cohorts (e.g. individuals with or without a movement disorder phenotype). All statistical analyses were performed using GraphPad Prism software, version 8.

### Standard protocol approvals, registrations, and patient consents

Caregivers reviewed the consent document prior to registration and survey completion. A waiver of documentation of informed consent was granted. This study was approved by the Stanford University Institutional Review Board.

## Supplementary Information

**Additional file 1**. **Table S1.** Likely pathogenic and pathogenic variants in *FOXG1*.

## Data Availability

The data that support the findings of this study are available from the corresponding author on reasonable request.

## Abbreviations

ACC: Augmentative and alternative communication
aCGH: Array comparative genomic hybridization
CVI: Cortical visual impairment
DNA: Deoxyribonucleic acid
