# Supplementary material for: Expanding genotype–phenotype correlations in FOXG1 syndrome: results from a patient registry
Source: Orphanet J Rare Dis. 2023 Jun 12;18:149. doi: 10.1186/s13023-023-02745-y (PMC10262363; doi:10.1186/s13023-023-02745-y)
Supplement: Supplementary file 1 — Additional file 1. Table S1. Likely pathogenic and pathogenic variants in FOXG1. [file 13023_2023_2745_MOESM1_ESM.docx]

**Supplemental Table 1. Novel (likely) pathogenic variants in *FOXG1***

| **Variant (c.)** | **Variant (p.)** | **Novel Variant** | **Segregation** | **ACMG** |
| --- | --- | --- | --- | --- |
| **Missense Variants** |  |  |  |  |
| c.553A>T | p.Ser185Cys | No | De Novo* | P |
| c.561C>G | p.Asn187Lys | No | De Novo* | P |
| c.563C>A | p.Ala188Glu | Yes | De Novo | LP |
| c.569T>A | p.Ile190Asn | No | De Novo | P |
| c.577G>A | p.Ala193Thr | No | De Novo | P |
| c.580A>T | p.Ile194Phe | Yes | De Novo | LP |
| c.581T>A | p.Ile194Asn | No | De Novo | P |
| c.584G>C | p.Arg195Pro | No | De Novo | P |
| c.619A>T | p.Ile207Phe | Yes | De Novo | LP |
| c.622T>C | p.Tyr208His | Yes | De Novo | LP |
| c.623A>G | p.Tyr208Cys | Yes | De Novo | LP |
| c.644T>C | p.Phe215Ser | Yes | De Novo* | LP |
| c.645C>G | p.Phe215Leu | Yes | Unknown | P |
| c.670G>A | p.Gly224Ser | Yes | De Novo | P |
| c.673T>C | p.Trp225Arg | Yes | De Novo | LP |
| **c.688C>T** | **p.Arg230Cys** | **No** | **De Novo** | **P** |
| **c.689G>A** | **p.Arg230His** | **No** | **De Novo*** | **P** |
| c.689G>T | p.Arg230Leu | Yes | De Novo* | LP |
| c.692A>G | p.His231Arg | Yes | De Novo | LP |
| c.695A>G | p.Asn232Ser | No | De Novo* | P |
| c.698T>C | p.Leu233Pro | Yes | De Novo | LP |
| **c.701C>T** | **p.Ser234Phe** | **Yes** | **De Novo** | **LP** |
| c.703C>T | p.Leu235Phe | No | De Novo | P |
| c.707A>T | p.Asn236Ile | Yes | Parental Mosaic | P |
| c.713G>A | p.Cys238Tyr | Yes | De Novo* | LP |
| c.731G>A | p.Arg244His | Yes | Unknown | LP |
| c.755G>A | p.Gly252Asp | No | De Novo* | P |
| c.757A>G | p.Asn253Asp | No | De Novo | P |
| c.758A>T | p.Asn253Ile | Yes | Parental Mosaic | P |
| c.763T>C | p.Trp255Arg | No | De Novo* | P |
| c.770T>G | p.Leu257Arg | Yes | De Novo | LP |
| c.800G>A | p.Gly267Asp | Yes | De Novo | P |
| c.811G>A | p.Gly271Ser | Yes | De Novo | P |
| c.824G>C | p.Arg275Pro | Yes | De Novo | LP |
| c.1178C>G | p.Ser393Trp | Yes | De Novo | LP |
| **Frameshift** |  |  |  |  |
| c.85_90delinsA | p.Gln29Argfs*90 | Yes | De Novo* | P |
| c.85delC | p.Gln29Argfs*163 | Yes | De Novo | P |
| c.108_109delinsG | p.Ser36Argfs*156 | Yes | Unknown | P |
| c.136dupC | p.Gln46Profs*75 | No | De Novo | P |
| c.177_186dupGCCGCCCGCC | p.Pro63Alafs*61 | Yes | De Novo* | P |
| c.222_223dupGC | p.Pro75Argfs*118 | No | Unknown | P |
| **c.256delC** | **p.Gln86Argfs*106** | **No** | **De Novo*** | **P** |
| **c.256dupC** | **p.Gln86Profs*35** | **No** | **De Novo** | **P** |
| c.265_266dupGG | p.Ala92Argfs*101 | Yes | Unknown | P |
| c.285delC | p.Asp95Glufs*97 | Yes | De Novo* | P |
| c.392delG | p.Gly131Alafs*61 | No | De Novo* | P |
| c.407_468del | p.Leu137Glyfs*297 | No | Unknown | P |
| c.460delG | p.Glu154Argfs*38 | No | De Novo* | P |
| **c.460dupG** | **p.Glu154Glyfs*301** | **No** | **De Novo** | **P** |
| c.479delG | p.Gly160Alafs*32 | No | De Novo* | P |
| c.501delG | p.Gly169Alafs*23 | Yes | De Novo | P |
| c.552delC | p.Phe184Leufs*8 | Yes | De Novo | P |
| c.938dupT | p.Leu314Profs*141 | Yes | De Novo | P |
| c.974_975insA | p.Ser326Glufs*129 | No | Parental Mosaic | P |
| c.1014dupC | p.Met339Hisfs*116 | Yes | Unknown | P |
| c.1021dupT | p.Tyr341Leufs*114 | Yes | De Novo | P |
| c.1060delC | p.His354Thrfs*9 | Yes | Unknown | P |
| c.1111dupG | p.Glu371Glyfs*84 | Yes | De Novo* | P |
| c.1120_1156del | p.Tyr374Profs*41 | Yes | De Novo | P |
| c.1160_1161delCGinsGTC | p.Ser387Cysfs*68 | Yes | De Novo | P |
| **Nonsense** |  |  |  |  |
| c.217C>T | p.Gln73Ter | Yes | De Novo | P |
| c.385G>T | p.Glu129Ter | No | De Novo* | P |
| c.430G>T | p.Glu144Ter | No | De Novo* | P |
| **c.537C>A** | **p.Tyr179Ter** | **No** | **De Novo** | **P** |
| c.586C>T | p.Gln196Ter | No | Unknown | P |
| **c.624C>G** | **p.Tyr208Ter** | **No** | **De Novo** | **P** |
| c.633delC | p.Met212Ter | Yes | De Novo | P |
| c.667C>T | p.Gln223Ter | Yes | De Novo | P |
| c.675G>A | p.Trp225Ter | Yes | De Novo | P |
| c.676C>T | p.Gln226Ter | Yes | De Novo | P |
| c.738C>G | p.Tyr246Ter | Yes | Unknown | P |
| c.762C>G | p.Tyr254Ter | No | De Novo* | P |
| c.920dupA | p.Tyr307Ter | Yes | Unknown | P |
| **c.924G>A** | **p.Trp308Ter** | **No** | **De Novo** | **P** |
| c.974_975delTG | p.Leu325Ter | Yes | De Novo* | P |
| **In-frame Deletion** |  |  |  |  |
| **c.515_577del** | **p.Gly172_Met192del** | **No** | **Parental Mosaic** | **P** |

*, reported *de novo* but parental testing report not available for confirmation

Bolded indicates that the variant is reported in >1 individual

LP = likely pathogenic, P = pathogenic
